# Practice Pattern Variation in Adoption of New and Evolving Percutaneous Coronary Intervention Procedures

**DOI:** 10.1155/2023/2488045

**Published:** 2023-05-04

**Authors:** Diana Naranjo, Jacob Doll, Charles Maynard, Kristine Beaver, Aasthaa Bansal, Christian D. Helfrich

**Affiliations:** ^1^Informatics, Decision-Enhancement and Analytic Sciences Center (IDEAS), VA Salt Lake City Health Care System, Salt Lake City, Utah, USA; ^2^Department of Internal Medicine, Division of Epidemiology, University of Utah School of Medicine, Salt Lake City, Utah, USA; ^3^Health Services Research & Development (HSR&D), Seattle-Denver Center of Innovation (COIN) for Veteran-Centered Value-Driven Care, US Department of Veterans Affairs (VA) Puget Sound Health Care System, Seattle, WA, USA; ^4^Department of Medicine, Division of Cardiology, University of Washington, Seattle, WA, USA; ^5^Department of Health Systems and Population Health, School of Public Health, University of Washington, Seattle, WA, USA; ^6^Department of Pharmacy, University of Washington, Seattle, WA, USA

## Abstract

**Objective:**

Assess factors contributing to variation in the use of new and evolving diagnostic and interventional procedures for percutaneous coronary intervention (PCI).

**Background:**

Evidence-based practices for PCI have the potential to improve outcomes but are variably adopted. Finding possible drivers of PCI procedure-use variability is key for efforts aimed at establishing more uniform practice.

**Methods:**

Veterans Affairs Clinical Assessment, Reporting, and Tracking Program data were used to estimate a proportion of variation attributable to hospital-, operator-, and patient-level factors across (a) radial arterial access, (b) intravascular imaging/optical coherence tomography, and (c) atherectomy for PCI. We used random-effects models with hospital, operator, and patient random effects. Overlap between levels generated cumulative variability estimates greater than 100%.

**Results:**

A total of 445 operators performed 95,391 PCI procedures across 73 hospitals from 2011 to 2018. The rates of all procedures increased over this time. 24.45% of variability in the use of radial access was attributable to the hospital, 53.04% to the operator, and 57.83% to patient-level characteristics. 9.06% of the variability in intravascular imaging use was attributable to the hospital, 43.92% to the operator, and 21.20% to the patient. Lastly, 20.16% of the variability in use of atherectomy was attributed to the hospital, 34.63% to the operator, and 57.50% to the patient.

**Conclusions:**

The use of radial access, intracoronary imaging, and atherectomy is influenced by patient, operator, and hospital factors, but patient and operator-level effects predominate. Efforts to increase the use of evidence-based practices for PCI should consider interventions at these levels.

## 1. Introduction

Percutaneous coronary intervention (PCI) has continued to see advances in its use, associated medications, therapies, and imaging in the last decade [1, 2]. Despite technological progress in PCI techniques and improved outcomes in complex coronary disease, varied application of specialized knowledge persists, such as intravascular imaging guidance [3, 4]. Medical practice variations have been documented across US regions, hospitals, and physicians since the 1980s [5–7] and suggest factors related to physician practice style or application of specialized knowledge and skills, may be a source of variation [8, 9]. Clinical decisions by a physician may drive adoption, as can hospital policies or trends in a particular geographic region, or a combination of these factors. Patient-level factors can also drive decisions, including patient preferences for treatment or risk tolerance [10]. Innovation adoption in healthcare techniques —the action of choosing to use something that is new or perceived as new with warranted increased use in clinical practice—is often unpredictable and its drivers remain elusive.

The Veterans Health Administration (VA) is a large national integrated healthcare system that captures procedure information for each PCI across the country through the Clinical Assessment, Reporting, and Tracking (CART) Program. In this work, we assessed 3 PCI techniques or adjunctive procedures with varying utilization among VA PCI programs: (a) radial artery access (TRA); (b) intravascular imaging via ultrasound (IVUS)/optical coherence tomography (OCT); and (c) atherectomy. Evidence to support these practices is variable, with strong evidence to support TRA over femoral access, emerging data supporting routine use of intravascular imaging for PCI, and mixed evidence supporting atherectomy. However, for each of these practices, substantial variability in practice has been observed.

The best way to promote rapid implementation of emerging and evolving technologies in the cardiac catheterization lab (cath lab), and among physicians in general, is unclear. Potential drivers of lagging best practice implementation are important to understand insomuch that without swift application, there could be inconsistent health outcomes and an undue impact on quality of care [11–16]. This study evaluates 3 potential drivers of clinical decision-making—hospital, operator, patient—to determine which might contribute most to procedure-use variability overall. These drivers encompass many other factors identified as major determinants of practice for cardiologist physicians. These include individual factors such as expertise, evidence and clinical guidelines, and institutional resources and policies [17].

## 2. Methods

We used data from the VA CART, which is a data repository for clinical care documentation and supports national reporting and quality improvement initiatives across VA hospitals. Operators enter procedures and patient characteristics into the CART system, such as preprocedure patient risk and indications as well as postprocedure outcomes for all coronary procedures performed in VA hospitals. The data entry elements are derived from the National Cardiovascular Data Registry (NCDR) and considered accurate and valid per ongoing independent assessment [18].

Through CART, we found all PCIs performed between 2011 and 2018 in VA hospitals across the US. This retrospective longitudinal analysis was based on a subpopulation of 450 operators who reported performing greater than or equal to 10 PCIs in any one year. These 450 operators collectively performed 95,391 interventions at 73 VA hospitals across the US. We performed a complete case analysis, excluding observations with missing data. The data analysis was performed between June 2020 and March 2021 [19]. The University of Washington and VA Internal Review Boards reviewed and determined the present study to be exempt from the need for oversight.

### 2.1. Measures

#### 2.1.1. Primary Outcome

Procedure-use indication captured in CART as a binary (yes/no) outcome for each of the three procedures, was the outcome of interest. The goal was to quantify how much of the variability in procedure use (yes vs. no) for each procedure was coming from the hospital, operator, or patient level.

#### 2.1.2. Primary Predictors

We assessed the following variables as potential drivers of practice pattern variation: (1) hospital, (2) operator (i.e., physician), and (3) patient. Each of these is uniquely identified in the data set. Variance is defined as a numerical value that indicates how widely units (e.g., individuals) in a group vary, and characterizes this difference from the group mean. Variance attributable to each of the primary predictors is our primary outcome in this investigation. We assessed the probability of procedure use: TRA, intracoronary imaging (IVUS and OCT) and atherectomy. These techniques were selected a priori due to their availability in CART and for being likely to vary in use due to hospital, physician, and patient-level factors. The use of TRA, IVUS and OCT, and atherectomy were identified through CART indicator variables for each procedure; indicator variables are derived from ICD-9 and ICD-10 procedure codes.

We included presence of cardiac surgery, an indication that the hospital could perform more complex procedures due to the availability of cardiothoracic surgery backup, as a hospital-level descriptive variable. Patient-level variables were obtained from CART and included sociodemographic characteristics such as patient age, sex, race, and ethnicity, indicators of cardiovascular health such as patient body mass index (BMI; normal, obese, overweight, or underweight), receipt of prior PCI, and receipt of prior coronary artery bypass graft surgery (CABG); additional indicators of health included the presence/absence of acute coronary syndrome, prior cardiogenic shock, cerebrovascular disease, chronic kidney disease, diabetes, congestive heart failure, hyperlipidemia, hypertension, prior myocardial infarction, and peripheral artery/vascular disease. Procedure priority, a variable indicating severity of disease and thus importance of the case (elective, elective staged, emergent, salvage, or urgent), was also included at the patient level. These descriptive variables at all levels—hospital, operator, and patient—were selected a priori based on their possible explanatory relationship with procedure use.

### 2.2. Statistical Analyses

We calculated proportions for all hospital, physician, and patient characteristics. We also calculated proportions of procedure-use counts over time for each procedure. We fit multilevel random-effects models to derive the probability of procedure use, account for in-hospital and by-physician clustering, and quantify the variance attributable to and within each level in our analysis: hospital (level 3), physician (level 2), and patient (level 1). We performed analyses at the procedure level. Log-link binomial generalized linear mixed models (GLMM) with robust variance estimators were used to model procedure use as a function of the covariates for each outcome. All models contained time as a fixed effect to account for secular trends in procedure use. We included random effects for hospital, physician, and patient in all the procedure-specific models. In total, there were 3 separate models with (1) TRA, (2) IVUS/OCT, and (3) atherectomy as the outcomes. The models provided variance estimates that were used to calculate the intra-class correlation coefficient, or variability, attributed to the random effect (i.e., cluster). Total variability as the sum of each—hospital, operator, and patient—variance component was expected to be either below or above 100%; a sum of 100% would indicate that we captured all variability perfectly. A sum of variance above 100% would indicate overlap between variance components in our models and sum below 100% would indicate that we had not incorporated enough of them. We encountered convergence challenges when attempting to fit the multilevel random effects models, likely due to overparameterization. The best strategy to achieve convergence consisted of running empty models with the hospital, physician, and patient levels; time remained a fixed effect. We used STATA 16 to conduct all analyses.

## 3. Results

**Figure 1:**
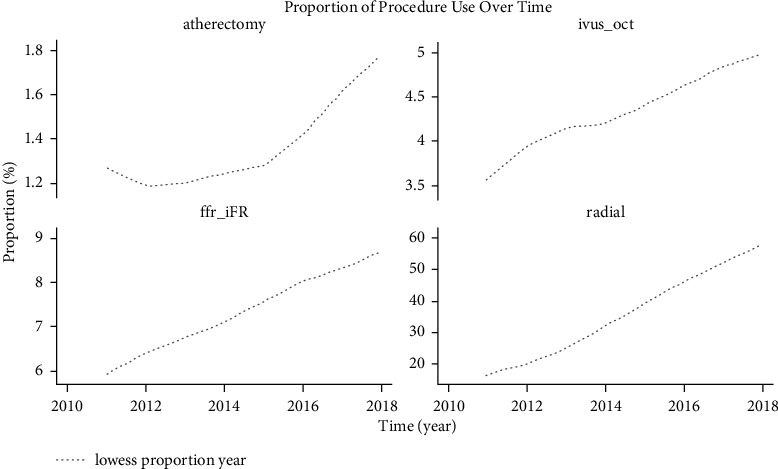
Relationship between proportion of procedure use and time. Procedure use increased each year 2011–2018; TRA (radial), IVUS, and OCT, and atherectomy show large increases in proportion over time. We applied a scatter plot smoothing tool called a locally weighted scatterplot smoothing (LOWESS) curve, which draws a curve through the plot as an aid to visualize trends, where it would otherwise be challenging to see a best fitting curve.

The VA patients who received the 95,391 procedures in our sample were predominantly male (98.5%), non-Hispanic (94.6%), and white (84.5%), belonging to BMI categories of overweight (34.9%) and obese (47.8%), with indications for hyperlipidemia (93.1%), hypertension (92.7%), and prior PCI (53.0%). Procedures tended to be elective (57.8%) or urgent (28.63%) and take place in hospitals with presence of cardiac surgery (68.99%) (Table 1). Overall use between 2011 and 2018 of TRA was 35.5%, IVUS and OCT were 4.3%, and atherectomy was 1.4%. Procedure use increased each subsequent year, with TRA, IVUS, and OCT, and atherectomy showing the largest increase in proportion over time (Figure 1). TRA was notably low in patients with prior CABG, 17.64% and 18.95%, respectively. Intravascular imaging was infrequently used even in high-complexity lesions such as left main (30.5%) and bifurcation (15.0%).

Patient-level factors predominated in the variability of atherectomy rates; patient- and operator-level factors were similarly important for radial access selection; and operator factors predominated for intravascular imaging. Hospital-level factors explained the least variability in each of the three procedures (Table 2).

## 4. Discussion

This study set out to quantify how much of the variability in procedure use might be attributed to the hospital, operator, or patient level in the VA healthcare system. The best way to promote new and evolving technologies in the cath lab, and among physicians in general, is unclear; in this descriptive analysis of potential care-level factors affecting innovation-adoption, we observed that operators could significantly affect the use of PCI innovations. Clinical decisions are expected to be primarily motivated by patient characteristics [20, 21]; however, given the observed operator-level contributions, further exploration into whether provider personal preferences play a more meaningful role appears warranted. It is possible, for example, that choices motivated by individual preference alone could determine a system's adoption of new procedures and a patient's access to evidence-based care. It is also possible that operator-level factors such as confidence or expertise might contribute to this preference, creating additional opportunities for education and lifelong learning. There are potentially more levels, or factors, contributing to these differences that we have not yet accounted for [17]. One example of this could be operators who also hold appointments at different healthcare systems (e.g., university medical centers); the other system's enforced practices might have an impact on an operator's choices at VA that we cannot measure. Our findings also corroborate the premise that all levels of care play some role and that there may be overlap between them.

The PCI innovations we evaluated have varying levels of support, which was actively accumulating over the course of our study period. Some have strong evidence for use, like vascular access site recommendations [13, 14, 22]. But there are some for which the evidence base is still being developed and/or might only be indicated for a small subset of patients, and only in cases of severe disease (e.g., atherectomies) [12, 15, 16, 23]. Even in the instances where there is strong evidence, such as radial-artery access site selection over femoral, there are situations where the benefits of using this approach must be balanced against competing procedural needs for improved guide support or complex patient anatomy [24]. Despite these clinical nuances, our summary data from over 95,000 procedures highlight marked variability attributable to physician decisions.

The type of procedure appeared to modify the level contributing most to overall procedure-use variability. Though speculative, it is possible that procedures with more limited and well-defined use cases (i.e., atherectomy for heavily calcified lesions) may display less operator-level variability than procedures with potentially broad use, such as intracoronary imaging. In addition, the relatively low contribution of hospital-level factors is interesting and may reflect the limited power of hospitals to influence individual physician decision-making. Prior studies have identified marked physician-level variation in PCI practice [25], and standardization of PCI best practices is a top priority of clinical societies [26]. Our study identifies 3 PCI techniques that may be amenable to interventions at the operator level.

There are several potential limitations of our work. We did not have measures for specific hospital or operator-level factors such as hospital leadership support or operator training [27, 28], that might meaningfully contribute to innovation adoption overall. Achieving complete model convergence proved challenging and required unanticipated modifications; the best strategy to achieve convergence consisted of running empty models with the hospital, physician, and patient levels. We would have preferred to adjust for the descriptive variables hypothesized to be associated with our variables of interest; however, the estimated values provide insight nonetheless, albeit potentially over or underestimating the relationship between our 3 levels and overall variance.

Further analysis of relative advantage, cost, and other characteristics pertaining to the evolving PCI technologies examined was beyond the scope of this study. The illustrated differences in adoption rate over time by type of innovation indicates that some of what drives adoption is likely unrelated to innovation characteristics and clinical efficacy; more research is needed to understand the interaction between specific innovation characteristics, such as cost, and each of the levels contributing to uneven adoption, including the ones we studied here and others we were unable to study. The cost of the equipment necessary to perform an atherectomy, for example, could be important to hospitals, but less relevant to providers or patients. Cost may be less of a factor in the national VA healthcare system, where patients face little or no out-of-pocket cost of cardiac procedures and physicians and hospitals have fewer financial incentives or penalties for care decisions relative to private hospitals in the US. Additionally, our study was unable to explore gender-related factors that may influence the decision-making process; although female Veterans represent the fastest growing population within the VA healthcare system, the data on which these findings are based from 2011 to 2018-- are not able to capture these nuances. In general, our use of the VA CART data limits the generalizability of our findings to other healthcare systems.

## 5. Conclusion

This study evaluated three potential drivers—hospital, operator, and patient—of procedure-use variability in VA PCI programs. Examining utilization of these procedures in this way allowed us to narrow down potential targets of future efforts to facilitate rapid implementation of best practices. The use of TRA, intracoronary imaging, and atherectomy is influenced by patient, operator, and hospital factors, but patient and operator-level effects predominate. Efforts to increase use of evidence-based practices for PCI should consider interventions at these levels.

## Figures and Tables

**Table 1:** Patient characteristics across procedure types 2011–2018 (*N* = 95,391).

Procedure type	Radial access (*n* = 29,218)	IVUS and OCT (*n* = 11,302)	Atherectomy (*n* = 4,811)	Total (*n* = 95,931)
Age (years) and mean (standard deviation)	67 (9)	67 (9)	70 (9)	66 (9)
Female	493 (17)	185 (1.6)	57 (1.2)	1519 (1.6)
Race			
American Indian or Alaskan Native	246 (0.8)	109 (1.0)	50 (1.0)	786 (0.8)
Asian	268 (0.9)	141 (1.2)	34 (0.7)	914 (1.0)
Black or African American	4759 (16.3)	1603 (14.2)	651 (13.5)	13,523 (14.2)
Native Hawaiian or other Pacific Islander	171 (0.6)	65 (0.6)	25 (0.5)	491 (0.5)
White	23,774 (81.7)	9384 (83.0)	4051 (84.2)	79,677 (83.5)
Hispanic ethnicity	1304 (4.5)	652 (5.8)	236 (4.9)	4867 (5.1)
Body mass index (kg/m^2^)			
Normal (18.5–24.9)	4228 (14.5)	1782 (15.8)	887 (18.4)	14,299 (15.0)
Underweight (<18.5)	163 (0.6)	76 (0.7)	36 (0.8)	563 (0.6)
Overweight (25.0–29.9)	9736 (33.3)	3993 (35.3)	1678 (34.9)	32,863 (34.4)
Obese (30.0–34.9)	14,765 (50.5)	5358 (47.4)	2171 (45.1)	46,387 (48.6)
Missing	326 (1.1)	93 (0.8)	39 (0.8)	1279 (1.3)
Prior percutaneous coronary intervention	13,987 (47.9)	6181 (54.7)	2570 (53.4)	49,043 (51.4)
Prior coronary artery bypass graft surgery	5154 (17.6)	2886 (25.5)	1433 (29.8)	27,515 (28.8)
Prior cardiogenic shock	422 (1.4)	217 (1.9)	92 (1.9)	1872 (2.0)
Cerebrovascular disease	6,002 (20.5)	2523 (22.3)	1343 (27.9)	21,887 (22.9)
Chronic kidney disease	6747 (23.1)	2839 (25.1)	1495 (31.1)	24,009 (25.2)
Diabetes	15,277 (52.3)	5872 (52.0)	2878 (59.8)	50,996 (53.5)
Congestive heart failure	8489 (29.0)	3912 (34.2)	2025 (42.1)	30,320 (31.8)
Hyperlipidemia	26,849 (91.9)	10,449 (92.4)	4518 (93.9)	88,467 (92.7)
Hypertension	27,057 (92.6)	10,434 (92.3)	4596 (95.5)	88,420 (92.7)
Peripheral artery disease	7396 (25.3)	3068 (27.2)	1795 (37.3)	25,785 (27.0)
Prior myocardial infarction	12,000 (41.1)	5182 (45.8)	2271 (47.2)	42,357 (44.4)
Indication			
Stable angina	8216 (28.6)	3413 (30.7)	1747 (37.1)	27,182 (28.5)
Unstable angina	5012 (17.4)	1982 (17.8)	837 (17.8)	15,814 (16.6)
Non-ST elevation myocardial infarction	6241 (21.7)	2333 (21.0)	800 (17.0)	20,507 (21.5)
ST elevation myocardial infarction	1162 (4.0)	584 (5.2)	56 (1.2)	5148 (5.4)
Atypical chest pain	3807 (13.2)	1140 (10.2)	446 (9.5)	11,337 (11.9)
Other	4295 (15.0)	1665 (15.0)	819 (17.4)	15,403 (16.1)
Procedure priority			
Elective-staged	1542 (5.3)	1058 (9.4)	721 (15.0)	1171 (1.2)
Elective	17,741 (60.7)	6400 (56.6)	2979 (61.9)	55,970 (58.7)
Emergent	1187 (4.1)	578 (5.1)	44 (0.9)	5386 (5.6)
Urgent	8394 (28)	3159 (28.0)	1020 (21.2)	27,161 (28.5)
Salvage	23 (0.1)	18 (0.2)	6 (0.1)	155(0.2)
Missing	331 (1.1)	89 (0.8)	41 (0.8)	5548 (5.8)
Prior treatment	2794 (9.6)	1541 (13.6)	368 (7.6)	9984 (10.5)
Left main disease	484 (1.7)	813 (7.2)	353 (7.3)	2662 (2.8)
Bifurcation lesion	2012 (6.9)	881 (7.8)	385 (8.0)	5857(6.1)
Graft lesion	1047 (3.6)	619 (5.5)	60 (1.2)	7871 (8.2)
Chronic total occlusion	1131 (3.9)	567 (5.0)	358 (7.4)	5431 (1.5)

IVUS/OCT; intravascular ultrasound/optical coherence tomography.

**Table 2:** Percent of total variance^*∗*^ attributable to hospital, physician, or patient.

Procedure	Level of care		
	Hospital (%)	Physician (%)	Patient (%)
Radial access	24.4	53.0	57.8
IVUS/OCT	9.1	43.9	21.2
Atherectomy	20.2	34.6	57.5

IVUS/OCT; intravascular ultrasound/optical coherence tomography. ^*∗*^Variance calculated as intraclass correlation (ICC)^+^ and reported as percentage. ^*+*^ICC=(var(*u*_0*j*_))/(var(*u*_0*j*_)+(*π*^2^/2))

## Data Availability

Data supporting the conclusions of the study are available to qualified personnel only at the US Department of Veterans Affairs.
